# Coronary plaque characteristics of acute myocardial infarction patients with and without previous ischemic stroke

**DOI:** 10.3389/fcvm.2026.1798730

**Published:** 2026-10-16

**Authors:** Qiuping Wang, Fuhong Dong, Chao Fang, Sining Hu, Haibo Jia, Weili Pan, Lulu Li, Jianjun Wu, Luping He, Yini Wang, Tao Chen, Huai Yu, Jiannan Dai, Bo Yu

**Affiliations:** 1 Harbin Medical University, Harbin, China; 2 Department of Cardiology, The Second Affiliated Hospital of Harbin Medical University, Harbin, China; 3 Key Laboratory of Myocardial Ischemia, Chinese Ministry of Education, Harbin Medical University, Harbin, China; 4 Department of Cardiology, the First Affiliated Hospital of Dalian Medical University, Harbin, China

**Keywords:** acute myocardial infarction, coronary plaque, ischemic stroke, optical coherence tomography, plaque vulnerability

## Abstract

**Background:**

Acute myocardial infarction (AMI) occurring after ischemic cerebral stroke leads to substantially worse short- and long-term outcomes, as indicated by emerging evidence. Few studies explore possible plaque features.

**Methods:**

From September 2019 to December 2021, 1050 AMI patients were enrolled. The individuals were divided into two groups based on whether they had suffered an ischemic stroke before percutaneous coronary intervention (PCI). Patients from both groups underwent 1:1 propensity score matching (PSM) with a caliper width of 0.02. Ultimately, the final study cohort comprised 386 patients.

**Results:**

Regarding culprit lesions, patients in the stroke group exhibited a greater prevalence of thin-cap fibroatheromas (TCFA, 70.0% vs. 55.4%, *p* = 0.003), macrophage accumulation (52.3% vs. 39.4%, *p* = 0.011), a longer lipid core length (15.3 ± 7.4 mm vs. 13.5 ± 6.4 mm, *p* = 0.013), and a higher lipid index (3,465.0 ± 2,135.8 vs. 2,946.4 ± 1,784.4, *p* = 0.011), compared to non-stroke patients. Concerning non-culprit plaques, prior stroke was significantly associated with a higher prevalence of TCFA (OR 2.08, 95% CI 1.15–3.74, *p* = 0.014). The clinical follow-up results revealed a significantly higher incidence of major adverse cardiovascular events in the stroke group (8.9% vs. 4.2%, *p* = 0.009, HR 2.61, 95% CI 1.28–5.34), along with a greater frequency of recurrent stroke (4.2% vs. 1.6%, *p* = 0.019, HR 3.78, 95% CI 1.23–9.30) and a higher cardiac mortality rate (5.8% vs. 1.6%, *p* = 0.011, HR 3.55, 95% CI 1.33–9.47) than the non-stroke group.

**Conclusion:**

In stroke patients, coronary multivessel disease is common and characterized by high-risk plaque features and increased cardiovascular event susceptibility, with prior ischemic stroke being associated with a higher prevalence of these OCT-defined high-risk features.

## 1 Introduction

Ischemic stroke (IS) is a clinical syndrome caused by occlusion of intracranial arteries, leading to ischemic necrosis of brain tissue (1). Cardiovascular and cerebrovascular diseases are leading causes of the global burden of disease and mortality (2). The coexistence of these two diseases is frequently observed. According to the most recent data, ischemic stroke accounts for 62.4% of all strokes (3). Acute ischemic stroke frequently results in multiple-organ dysfunction, with heart damage being the primary factor exacerbating the condition (4). Amarenco and colleagues (5) reported that 61.9% of patients who experienced non-fatal ischemic stroke had evidence of coronary atherosclerotic plaque, while 25.7% exhibited coronary stenosis. The incidence of composite events (including recurrent stroke, myocardial infarction, and vascular death) within one year following a stroke has been documented to be 24.7% (6). Boulanger et al. (7) demonstrated that although patients with previous stroke were treated according to secondary prevention guidelines, 25% of patients still had cardiovascular events, and the risk of recurrent stroke was higher. Existing evidence indicates that stroke is an independent predictor of poor prognosis in patients with myocardial infarction (8). Optical coherence tomography (OCT) (9) has unique advantages in plaque assessment and characterization of plaque composition, and has been widely used in the interventional diagnosis and treatment of coronary artery disease. The study aims to assess coronary plaque characteristics using OCT imaging in individuals with previous ischemic stroke and to investigate the association between ischemic stroke history and clinical outcomes in patients with AMI.

## 2 Methods

### 2.1 Study design and population

A total of 1,050 patients with AMI underwent OCT examinations at the Department of Cardiology of the Second Affiliated Hospital of Harbin Medical University from September 2019 to December 2021. All participants were categorized into two groups according to the history of ischemic stroke: the stroke group (*n* = 193) and the non-stroke group (*n* = 857). Previous research indicated that variables such as age, gender, hypertension, diabetes mellitus, hyperlipidemia, and smoking influence plaque load and characteristics (10–13). Hence, these variables were utilized for 1:1 propensity score matching (PSM) based on the clinical data from both groups, with a caliper set at 0.02. A total of 193 pairs of patients, amounting to 386 patients, were successfully matched. This included 193 patients in the stroke group and 193 in the non-stroke group. Coronary angiography and OCT images were retrospectively analyzed.

Patients with clinical instability or coronary anatomical characteristics that could compromise the safety, feasibility, or reliability of OCT acquisition were excluded. These conditions included left main coronary artery disease, chronic total occlusion, severe vessel tortuosity or calcification preventing OCT guidewire advancement, and high thrombus burden that might interfere with accurate plaque visualization. In addition, patients with poor OCT image quality, including inadequate blood clearance, insufficient signal penetration, or significant imaging artifacts, were excluded according to predefined OCT analyzability criteria. Therefore, only patients with stable clinical conditions and interpretable OCT images were included for plaque analysis. Ultimately, 386 patients were enrolled, with 193 in the stroke group and 193 in the non-stroke group. The study flow chart is shown in Figure 1.

**Figure 1:**
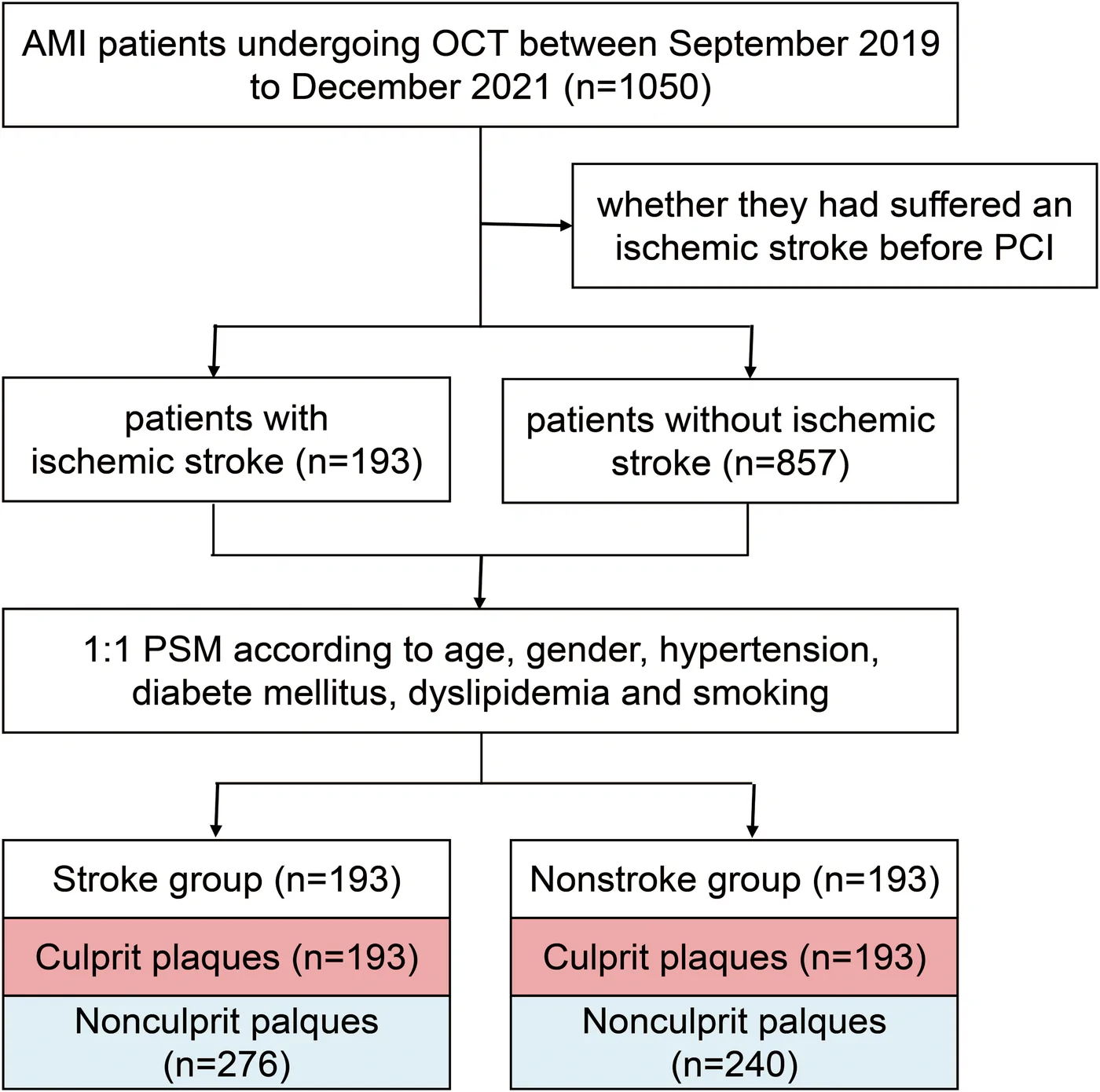
Study flow chart.

The definition of AMI was based on the 2018 European Society of Cardiology Guidelines for the management of AMI (14). The definition of ischemic stroke was based on the 2020 Canadian secondary prevention guidelines for stroke (15). Plaque measurements for culprit and non-culprit lesions were performed according to previously established guidelines and consensus (9). A lipid-rich plaque was defined as a plaque with a lipid arc >90°. Lipid core length was defined as the length of a plaque with a lipid arc >90° measured on the longitudinal view. The lipid index was defined as the lipid core length multiplied by the mean lipid arc. The fibrous cap thickness of each lipid-rich plaque was measured three times on the thinnest part, and the mean value was taken. A TCFA was defined as a lipid-rich plaque (lipid pool arc ≥180°) with a thin fibrous cap (thinnest fibrous cap thickness <65 μm). A microchannel was defined as a tubular or porous signal-poor structure with clear boundaries and a diameter of approximately 50–300 μm that could be observed in ≥3 consecutive frames. Cholesterol crystals were defined as signal-rich linear structures typically located in the necrotic core of lipid-rich plaques or adjacent to the fibrous caps. Macrophages appeared as high-attenuation, high-brightness, band-like or dot-like structures on OCT images, typically accompanied by back reflections. Other specific diagnostic criteria can be found in the Supplementary Material.

The primary endpoint of the study is MACE, defined as cardiogenic death, non-fatal myocardial infarction, ischemia-induced revascularization (target/nontarget vessels). The secondary endpoints include the components of the primary endpoint along with angina, heart failure admission, and recurrent stroke, and bleeding events, specifically focusing on type 2–5 bleeding events requiring hospitalization or intervention, as defined by the Association for Academic Research on Bleeding (16). Detailed definitions of the events included in the primary and secondary endpoints are provided in the Supplementary Material.

This study was conducted in accordance with the principles outlined in the Declaration of Helsinki and received approval from the Institutional Review Board of the Second Affiliated Hospital of Harbin Medical University. All patients provided written informed consent. We followed up with patients via phone, outpatient visits, or during hospitalization to collect patient information. We retrieved patients’ clinical information using a computerized system.

### 2.2 OCT image acquisition and analysis

Before interventional therapy, all patients received aspirin 300 mg and either clopidogrel 300 mg or ticagrelor 180 mg. Aspiration thrombectomy was performed to reduce the thrombus burden prior to OCT imaging. In this study, OCT images of all patients were acquired using an ILUMIEN OPTIS OCT imaging system (St. Jude Medical, St. Paul, MN, USA). Two experienced researchers analyzed OCT data offline using a professional OCT image analysis system (Light Lab Imaging Offline Review Workstation). Discrepancies in OCT image analysis between the two researchers were resolved by consulting a third researcher. The investigator performing the OCT image analysis was blinded to the patients’ clinical characteristics.

All OCT examinations in this study were imaged using time-domain OCT (M2/M3 system, Saint Jude Medical, Westford, MA, USA) or frequency-domain OCT (C7 Dragonfly, Saint Jude Medical, St Paul, MN, USA). The OCT examination of culprit lesions was performed after the restoration of antegrade coronary blood flow and once the patient had achieved hemodynamic stability. Meanwhile, non-coronary vascular OCT imaging was performed after the reconstruction of blood flow in culprit lesions. If interventions such as predilation or rotational atherectomy were performed on the lesion before OCT examination, such patients were excluded from the study. When using time-domain OCT to capture images, the OCT-blocking balloon catheter was sent to the distal end of the lesion site, and the OCT imaging guide wire was inserted into the OCT-blocking balloon catheter. The blocking balloon was inflated to a pressure of 0.4–0.6 atm, and the OCT imaging guide wire was automatically retracted at a speed of 1–3 mm/s. After imaging was completed, the blocking balloon was rapidly deflated by applying negative pressure. For frequency-domain OCT imaging, the imaging guidewire automatically retracted at a speed of 20 mm/s, while contrast agent was continuously injected through a guide catheter at 3–4 mL/s.

### 2.3 Statistical method

All data were analyzed by independent statisticians in the core laboratory. Patients in the two groups were matched 1:1 by propensity score matching (PSM) for age, gender, hypertension, diabetes, dyslipidemia, smoking, and alcohol consumption, with a caliper set at 0.02. The Kolmogorov–Smirnov test was utilized to assess the normality of the data distribution. Non-normally distributed measurement data were presented as the median and upper and lower quartiles and compared between groups using the Mann–Whitney U test. Count data were presented as frequencies and percentages and compared between groups using the chi-square test. If the chi-square test assumptions were not met, Fisher's exact probability test was employed. Ranked data were presented as frequencies and percentages. Due to the clustering effect of multiple non-culprit atherosclerotic plaques in the same patient, the data for the non-culprit plaques were analyzed using generalized estimating equations and stepwise logistic regression analysis to identify independent risk factors for the stroke group. For non-culprit atherosclerotic plaques, regression models and a working correlation matrix were chosen based on the Quasi-likelihood Information Criterion (QIC) principles, with the final selection being the model with the smallest QIC value. To address multicollinearity, the stepwise regression method was used for variable screening, excluding variables without statistical significance. The independent variables include preadmission medication (including antiplatelet drugs, statins, ACEI/ARB, β-blockers, CCB drugs), TCH, TG, HDL-C, LDL-C, ApoA, and ApoB. A *P*-value of <0.05 was considered statistically significant. All statistical analyses were conducted using SPSS version 25.0 (IBM Corp., Armonk, NY, USA).

## 3 Results

### 3.1 Baseline characteristics

This section summarizes the clinical data of the two groups after PSM. The data indicated no significant differences in age, sex, or risk factors for coronary heart disease between the two groups (Table 1). In the stroke group, a higher proportion of patients had previously taken medications such as antiplatelet drugs, statins, and β-blockers. Compared to the non-stroke group, the stroke group had lower total cholesterol [TC, 180.1 (156.0–202.8) mg/dL vs. 166.1 (138.4–194.1) mg/dL, *p* = 0.006] levels and low-density lipoprotein cholesterol [LDL-C, 113.6 (86.0–138.8) mg/dL vs. 98.6 (75.6–116.7) mg/dL, *p* < 0.001] levels, as well as higher creatinine levels [85.0 (73.0–95.0) mg/dL vs. 88.0 (76.0–107.0) mg/dL, *p* = 0.028]. Clinical data before propensity score matching (PSM) are presented in Supplementary Table S1.

**Table 1:** Comparison of baseline characteristics of the stroke group and non-stroke group.

Variables	Non-stroke group (*n* = 193)	Stroke group (*n* = 193)	*P*-value
**Clinical data**			
Age, years	65.0 (56.0–71.0)	64.0 (58.0–72.0)	0.536
Male	141 (73.1)	139 (72.0)	0.820
BMI, kg/m^2^	25.2 (22.6–27.5)	25.3 (22.5–27.8)	0.505
Current smoker	87 (45.1)	75 (38.9)	0.216
Current drinker	36 (18.7)	34 (17.6)	0.792
Family history of CAD	122 (63.2)	126 (65.3)	0.671
Hypertension	60 (31.1)	52 (26.9)	0.370
Diabetes mellitus	52 (26.9)	54 (28.0)	0.820
Dyslipidemia	58 (52.7)	52 (47.3)	0.499
**Laboratory data**			
HbAlc, %	6.6 (5.6–7.2)	6.4 (5.6–6.8)	0.579
hsCRP, mg/L	7.6 (2.7–13.3)	7.7 (2.7–12.6)	0.919
TC, mg/dL	180.1 (156.0–202.8)	166.1 (138.4–194.1)	0.006
Triglyceride, mg/dL	142.7 (80.5–165.9)	138.2 (84.5–169.0)	0.786
HDL-C, mg/dL	44.9 (37.5–52.2)	45.6 (38.3–53.6)	0.331
LDL-C, mg/dL	113.6 (86.0–138.8)	98.6 (75.6–116.7)	<0.001
ApoA, g/L	1.2 (1.0–1.3)	1.2 (1.00–1.3)	0.984
ApoB, g/L	1.0 (0.8–1.2)	0.94 (0.7–1.3)	0.081
Crea, mg/dL	85.0 (73.0, 95.0)	88.0 (76.0, 107.0)	0.028
**Previous medication use**			
Antiplatelet drugs	70 (36.3)	122 (63.2)	<0.001
Statins	19 (9.8)	50 (25.9)	<0.001
ACEI/ARB	15 (7.8)	27 (14.0)	0.050
β-blocker	17 (8.8)	37 (19.2)	0.003
CCB	38(19.7)	45(23.3)	0.386

Data are presented as median (interquartile range) or number (%).

ACEI, angiotensin-converting enzyme inhibitors; ApoA, Apolipoprotein A; ApoB, Apolipoprotein B; ARB, angiotensin receptor blocker; CAD, coronary artery disease; CCB, calcium channel blockers; CKD, Chronic kidney disease; Cr, creatinine; HbAlc, hemoglobinA1c; HDL-C, high-density lipoprotein cholesterol; hs-CRP, high-sensitive C-reactive protein; LDL-C, low-density lipoprotein cholesterol; TC, total cholesterol.

### 3.2 OCT findings

OCT images of the stroke group revealed a total of 367 coronary vessels, comprising 193 culprit plaques and 276 non-culprit plaques. The non-stroke group presented a total of 443 coronary vessels, including 193 culprit plaques and 219 non-culprit plaques. Statistical analysis revealed no significant difference in culprit lesion types between the two groups (*p* = 0.086). Compared with the non-stroke group, culprit plaques in the stroke group exhibited a higher prevalence of thin-cap fibroatheroma (TCFA, 70.0% vs. 55.4%, *p* = 0.003), macrophage accumulation (52.3% vs. 39.4%, *p* = 0.011), cholesterol crystals (35.2% vs. 21.8%, *p* = 0.003), and a higher proportion of mixed (red and white) thrombus (16.6% vs. 13.0%, *p* = 0.019 for overall thrombus type distribution) (Table 2). Additionally, culprit plaques in the stroke group exhibited a longer lipid core length (15.3 ± 7.4 mm vs. 13.5 ± 6.4 mm, *p* = 0.013) and a higher lipid index (3,465.0 ± 2,135.8 vs. 2,946.4 ± 1,784.4, *p* = 0.011) compared to the non-stroke group. No significant differences were observed in other OCT parameters, including minimal lumen area, percent area stenosis, fibrous cap thickness, and calcification (*p* = 0.098).

**Table 2:** Comparison of coronary angiography and optical coherence tomography characteristics of culprit plaques.

Variables	Non-stroke group (*n* = 193)	Stroke group (*n* = 193)	*P*-value
CAG characteristics			
Diagnosis			0.110
STEMI	147 (76.17)	133 (68.91)	
NSTEMI	46 (23.83)	60 (31.09)	
Pre-PCI TIMI flow			0.097
0	15 (7.8)	16 (8.3)	
1	27 (14.0)	40 (20.7)	
2	118 (61.1)	118 (61.1)	
3	33 (17.1)	19 (9.8)	
Aspiration thrombectomy	137 (71.0)	138 (71.5)	0.910
time from symptom onset to angiography	29.0 (20.0, 38.0)	29.5 (20.0, 36.0)	0.470
OCT characteristics			
Culprit vessels			0.138
LAD	99 (51.3)	80 (41.5)	
RCA	63 (32.6)	79 (40.9)	
LCX	31 (16.1)	34 (17.6)	
Culprit lesion type			0.086
Plaque rupture	125 (64.8)	139 (72.0)	
Plaque erosion	34 (17.6)	37 (19.2)	
Calcified nodule	7 (3.6)	3 (1.6)	
Others	27 (14.0)	14 (7.3)	
Lesion length, mm	24.8 ± 9.0	24.3 ± 8.9	0.056
MLA，mm^2^	1.3 ± 0.8	1.4 ± 0.8	0.499
Area stenosis, %	80.7 ± 10.3	79.2 ± 10.3	0.183
Lipid-rich plaque	184 (95.3)	189 (97.9)	0.158
Lipid core length, mm	13.5 ± 6.4	15.3 ± 7.4	0.013
Mean lipid arc,°	207.9 (172.7, 244.2)	216.3 (182.1, 251.3)	0.084
Maximum lipid arc,°	360.0 (284.2, 360.0)	360.0 (301.4, 360.0)	0.273
FCT, μm	60.0 (43.3, 83.3)	60.0 (43.3, 73.3)	0.585
Lipid core index	2,946.4 ± 1,784.4	3,465.0 ± 2,135.8	0.011
TCFA, %	107 (55.4)	135 (70.0)	0.003
Cholesterol crystal	42 (21.8)	68 (35.2)	0.003
Macrophages	76 (39.4)	101 (52.3)	0.011
Microchannel	38 (19.7)	51 (26.4)	0.116
Thrombus			0.019
None	19 (9.8)	33 (17.1)	
Red	123 (63.7)	116 (60.1)	
White	26 (13.5)	12 (6.2)	
Red and white	25 (13.0)	32 (16.6)	
Calcification	32 (16.6)	45 (23.3)	0.098
Microcalcification	83 (43.0)	93 (48.2)	0.307

Data are presented as number (%) or median (interquartile range).

FCT, fibrous cap thickness; MLA, minimal lumen area; TCFA, thin-cap fibroatheroma.

Table 3 provides an overview of the patient-level OCT analysis of non-culprit plaques. Relative to the non-culprit plaques in the non-stroke group, those in the stroke group exhibited a higher incidence of TCFA (OR 2.08; 95% CI 1.15–3.74; *p* = 0.014).

**Table 3:** The predictive value of stroke for TCFA in culprit plaque and nonculprit plaque.

Stroke	OR	95%CI	*P*-value
Culprit plaque - level	1.87	1.23–2.84	0.003
Nonculprit plaque - level	2.08	1.15–3.74	0.014

CI, confidence interval; OR, odd ratio.

Qualitative OCT findings regarding the features of non-culprit and culprit plaques revealed that patients with a history of stroke had a higher prevalence of TCFA and macrophage accumulation (*p* = 0.003 and *p* = 0.011). There was no significant difference in other plaque characteristics between the two groups (Figure 2A). In contrast, results revealed that patients with a history of stroke were more likely to have culprit lesions with TCFA, macrophages, calcification, and cholesterol crystals (*p* = 0.022, *p* = 0.019, *p* < 0.001, and *p* = 0.001) compared to patients without a history of stroke (Figure 2B). In culprit plaques, patients with a history of stroke had a numerically greater mean lipid arc than those without a history of stroke, although the difference was not statistically significant (*p* = 0.084; Figure 3A). The lipid core index was significantly higher in patients with a history of stroke (*p* = 0.011; Figure 3B).

**Figure 2:**
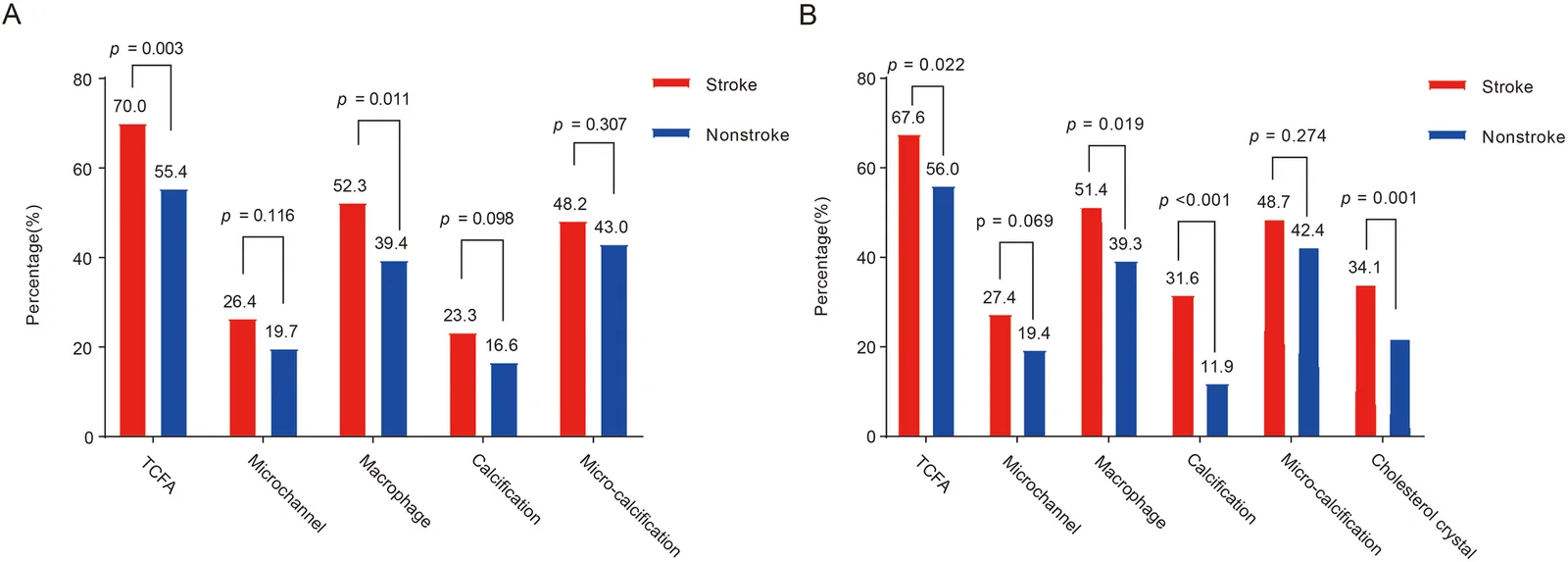
Qualitative OCT findings regarding plaque features of nonculprit plaques and culprit plaques. **(A)** revealed that patients with a history of stroke had a higher prevalence of TCFA and macrophages. There was no significant difference in the remaining plaque characteristics between the two groups. **(B)** revealed that patients with a history of stroke were more likely to have culprit lesions with TCFA, cholesterol crystals, microchannels, and calcifications compared to patients without a history of stroke. TCFA, thin-cap fibroatheroma.

**Figure 3:**
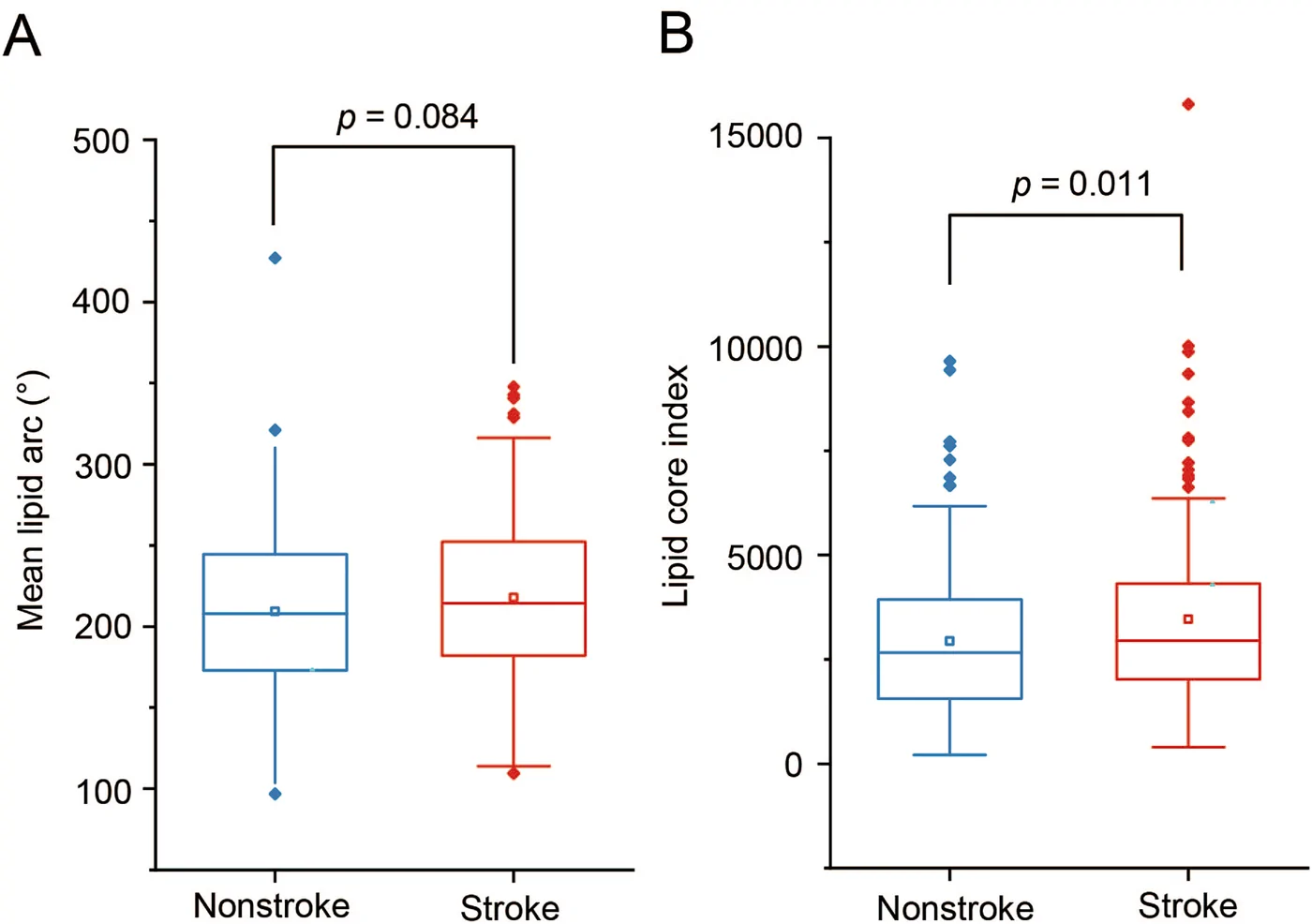
Comparison of mean lipid arc and lipid core index in culprit plaques between the stroke and non-stroke groups. **(A)** The mean lipid arc was numerically greater in the stroke group, but the difference was not statistically significant (*p* = 0.084). **(B)** The lipid core index was significantly higher in the stroke group (*p* = 0.011).

TCFA is an important marker of vulnerable plaques. To determine whether stroke is an independent risk factor for TCFA, logistic regression analysis was used for culprit plaques, and generalized estimating equations with stepwise regression were applied to non-culprit plaques (Table 3). In both culprit and non-culprit plaques, stroke was independently associated with TCFA (culprit plaques: *p* = 0.003, OR 1.87, 95% CI 1.23–2.84; non-culprit plaques: *p* = 0.014, OR 2.08, 95% CI 1.15–3.74). A study employing univariate analysis and multivariate logistic regression demonstrated that stroke is an independent risk factor for TCFA and a predictor of vulnerable plaques (Supplementary Table S2).

### 3.3 Clinical outcomes

Results indicated that a total of 382 patients completed the clinical follow-up with a duration of 18 months; the cohort comprised 191 patients in the non-stroke group and 191 in the stroke group. The stroke group showed a higher incidence of major adverse cardiovascular events [MACE, 8.9% vs. 4.2%, *p* = 0.009, HR 2.61 (95% CI 1.28–5.34)], recurrent stroke [4.2% vs. 1.6%, *p* = 0.019, HR 3.78 (95% CI 1.23–9.30)], and cardiogenic death [5.8% vs. 1.6%, *p* = 0.011, HR 3.55 (95% CI 1.33–9.47)] than the non-stroke group. There was no statistically significant difference in other endpoint events, including death from other causes, recurrent myocardial infarction, readmission for angina or heart failure, revascularization, and bleeding (Table 4). Kaplan–Meier curves for the clinical outcomes after excluding patients lost to follow-up showed significant differences between the groups in the cumulative 18-month incidence of MACE, recurrent stroke, and cardiogenic death (Figures 4A–C).

**Table 4:** Clinical prognosis analysis of non-stroke group and stroke group.

Variables	Non-stroke group (*n* = 191)	Stroke group (*n* = 191)	HR (95%CI)	*P-*value
MACE	8 (4.2)	17 (8.9)	2.61 (1.28∼5.34)	0.009
Recurrent stroke	3 (1.6)	8 (4.2)	3.78 (1.23∼9.30)	0.019
Cardiogenic death	3 (1.6)	11 (5.8)	3.55 (1.33∼9.47)	0.011
Death for other causes	1 (0.5)	3 (1.6)	3.02 (0.31∼29.07)	0.338
Recurrent MI	2 (1.1)	3 (1.6)	1.50 (0.25∼8.96)	0.659
Readmission for angina or heart failure	7 (3.7)	13 (7.0)	1.89 (0.75∼4.73)	0.175
Revascularization	4 (2.1)	5 (2.6)	2.01 (0.60∼6.67)	0.255
Bleeding	4 (2.1)	4 (2.1)	1.00 (0.25∼4.00)	0.999

Data are presented as number (%).

MACE, Major adverse cardiovascular event; MI, Myocardial infarction.

**Figure 4:**
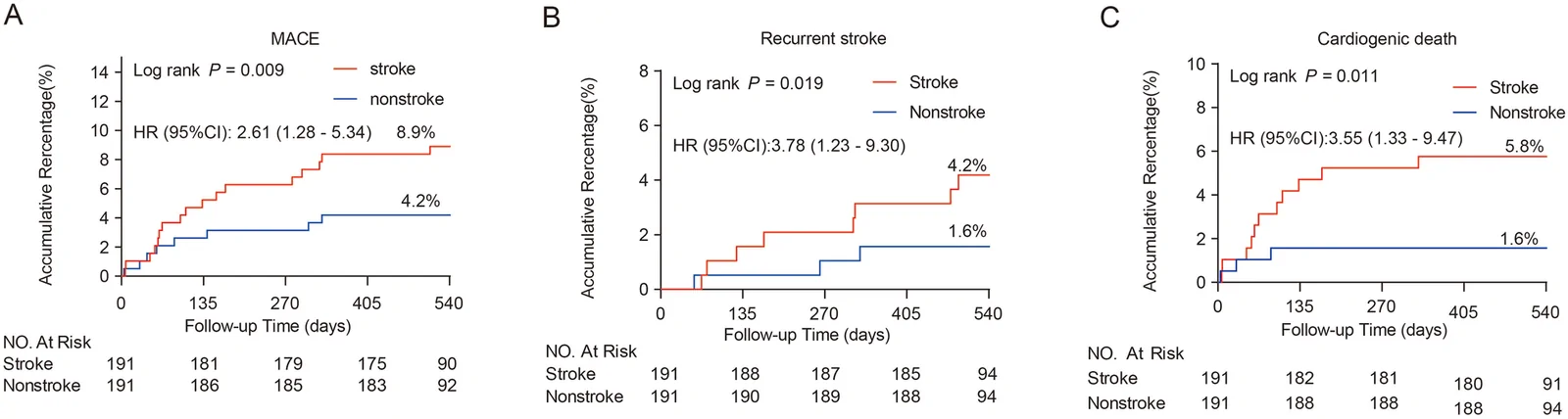
Kaplan–meier curves showing cumulative incidence of MACE **(A)**, recurrent stroke **(B)**, and cardiogenic death **(C)** hazard ratios were calculated using cox proportional hazards models. Log-rank tests were used for comparison between groups. CI, confidence interval; HR, hazard ratio; MACE, major adverse cardiovascular event; NO. At Risk, number at risk.

## 4 Discussion

The primary findings of the study are outlined below: (1) Patients with AMI with a history of stroke exhibited a higher prevalence of multi-vessel changes, TCFA, and macrophages in both culprit and non-culprit plaques compared to those without such a history. (2) The cumulative incidence at 18-month of MACEs, recurrent stroke, and cardiogenic death in the ischemic stroke group was higher than in the non-stroke group.

### 4.1 Plaque characteristics of culprit and nonculprit lesions in AMI patients with a stroke history

To date, no studies have utilized OCT to systematically examine the association between prior ischemic stroke and coronary plaque characteristics, or to investigate their relationship with subsequent cardiovascular events. However, the link between cerebrovascular disease, systemic atherosclerosis, and adverse outcomes is well established (17–19). For instance, intracranial vascular studies have identified several vulnerability features, including intraplaque hemorrhage, a lipid-rich necrotic core, a thin or ruptured fibrous cap, plaque inflammation, neovascularization, plaque volume, and irregular luminal surface morphology as hallmarks of high-risk lesions (20). In the coronary territory, the CLIMA study further demonstrated that a composite indicator composed of minimum lumen area, fibrous cap thickness, lipid arc circumference, and macrophage presence was associated with an approximately 7.5-fold higher risk of cardiac death and myocardial infarction within one year (21, 22). Building upon this established knowledge, this study expands upon existing evidence by providing detailed OCT-based characterization of plaque phenotypes specifically in AMI patients with and without a history of ischemic stroke, thereby offering a more granular understanding of how prior cerebrovascular events may correlate with coronary vulnerability.

Our study found that patients with AMI who had a previous stroke exhibited a higher prevalence of inflammation and vulnerable characteristics in both culprit and non-culprit plaques compared to those who did not have a stroke. These findings are consistent with prior research. Following an ischemic stroke, the body undergoes persistent inflammation mediated by stem cells. Recent studies have shown that, compared to stable plaques, the cellular tissues of stroke patients contain mRNA that can trigger inflammation and degrade the key components that prevent plaque rupture (23). These observations corroborate the conclusions of the CLIMA studies.

Notably, we found that the density of cholesterol crystals in the culprit plaques was higher in the stroke group. Cholesterol crystals are not only markers of atherosclerosis but are also associated with coronary artery inflammation and vulnerability (21, 22). Cholesterol crystallization results from the accumulation of free cholesterol and the disruption of cholesterol homeostasis in macrophages. Excess esterified cholesterol and crystals are released from dead foam cells into the extracellular space, where they manifest as cholesterol crystals in coronary plaque. Our research confirms that the presence of cholesterol crystals in plaques is associated with poorer clinical outcomes. This finding is consistent with a recent study showing that the presence of cholesterol crystals predicts poorer clinical outcomes within one year (24).

Our research indicates that the prevalence of calcification in the plaques of stroke patients is relatively high. Although calcification is not a marker of plaque vulnerability, calcified lesions may affect stent expansion (25, 26).

### 4.2 Prior stroke was associated with a higher prevalence of OCT-defined high-risk plaque features

TCFA rupture is the main pathological mechanism of ACS, and the early identification of TCFA is crucial for preventing myocardial infarction. However, large-scale imaging studies such as PROSPECT have revealed that the positive predictive value of using plaque morphological features such as TCFA to predict future events is limited (15%–25%) (27–29), highlighting the necessity of integrating additional clinical characteristics to optimize risk stratification. In this observational cohort, we observed an association between a history of ischemic stroke and a higher prevalence of vulnerable plaques. This history may act as a readily available marker of heightened systemic atherosclerotic burden and residual vascular risk. Clinically, this association may help identify AMI patients with prior cerebrovascular events who have a higher burden of systemic atherosclerotic risk and may require more intensive secondary prevention strategies. Whether advanced imaging approaches can improve risk stratification or clinical management requires further prospective investigation.

## 5 Limitations

The study has the following limitations. Firstly, selection bias is inherent to our retrospective, single-centre design, stemming from the inclusion of only AMI patients with analyzable OCT images, which may limit generalizability. Despite propensity score matching, residual confounding cannot be entirely ruled out due to unmeasured factors such as inflammatory status and medication usage, potentially affecting our effect estimates. Secondly, data regarding specific details of prior stroke events, including subtypes, severity, and time intervals between events and AMI, were unavailable, thereby precluding further subgroup analysis. Thirdly, OCT evaluation was performed solely at baseline; the absence of follow-up imaging precluded the assessment of longitudinal plaque feature evolution. Additionally, despite efforts to minimize confounding variables using propensity scores and linear regression analysis, it was not possible to achieve balance across all factors, including education levels and inflammation.

## 6 Conclusions

AMI patients with a stroke history exhibited a greater burden of high-risk plaque characteristics in both culprit and non-culprit lesions than those without, and these findings were associated with a greater prevalence of OCT-defined high-risk features and suggested a potentially poorer prognosis.

## Data Availability

The original contributions presented in the study are included in the article/Supplementary Material, further inquiries can be directed to the corresponding author.
