# Supplementary material for: Coronary plaque characteristics of acute myocardial infarction patients with and without previous ischemic stroke
Source: Front Cardiovasc Med. 2026 Oct 16;13:1798730. doi: 10.3389/fcvm.2026.1798730 (PMC13719401; doi:10.3389/fcvm.2026.1798730)
Supplement: Supplementary file 1 [file Datasheet1.docx]

**Supplemental Materials**

1. Diagnostic criteria for clinical outcomes:

STEMI is characterized by chest pain lasting more than 30 minutes and presentation to the hospital within 12 hours of symptom onset. Diagnosis is confirmed by a 12-lead electrocardiogram demonstrating at least two consecutive leads with ST-segment elevation greater than 0.1 mV or the presence of a new left bundle branch block. Additionally, elevated levels of blood markers for myocardial injuries, such as creatine kinase isoenzyme MB (CK-MB) or cardiac troponin T/I (cTnT/I), are observed. NSTEMI is identified by the presence of ischemic chest pain symptoms, the absence of significant ST-segment elevation on electrocardiogram, and elevated blood myocardial injury markers (CK-MB or cTnT/I). The definition of ischemic stroke is based on the 2020 Canadian Guidelines for Secondary Prevention of Stroke (1), which states that when there is evidence of responsible ischemic lesions on neuroimaging, stroke can be diagnosed regardless of the duration of symptoms/signs. However, when imaging evidence of responsible lesions cannot be obtained, the time limit for diagnosing stroke is still based on the duration of symptoms/signs exceeding 24 hours. The diagnostic criteria include: According to the definition of ischemic stroke in the International Classification of Diseases (ICD-11), when neuroimaging shows a responsible ischemic lesion, the diagnosis of ischemic stroke can be made regardless of the duration of symptoms or signs. However, when imaging evidence of the responsible lesion is unavailable, the diagnosis is still based on symptoms or signs lasting more than 24 hours. It should be noted that most TIA patients have symptoms lasting no more than 0.5 to 1 hour. 1. Acute onset; 2. Focal neurologic deficits (e.g., unilateral facial or limb weakness or numbness, speech impairment, etc.), and in a few cases, global neurologic deficits; 3. Imaging showing a responsible lesion or symptoms/signs persisting for more than 24 hours; 4. Exclusion of non-vascular causes; 5. Brain CT/MRI ruling out intracerebral hemorrhage.

Cardiogenic shock is a clinical syndrome characterized by a series of symptoms such as ischemia, hypoxia, metabolic disorders, and damage to vital organs, which occurs when cardiac function is compromised due to various causes, resulting in a significant reduction in cardiac output, a decrease in blood pressure, and severe insufficient perfusion of vital organs and tissues, leading to systemic microcirculatory dysfunction.

bleeding events, specifically focusing on type 2-5 bleeding events that require hospitalization intervention as defined by the Association for Academic Research on Bleeding (2) and details as follows:

Type 0: No bleeding.

Type 1: Non-active bleeding, patients do not need to seek medical attention or hospitalization (such as bruising, hematoma, hemorrhoids bleeding, but not limited to these conditions); patients self-stop medication due to bleeding without consulting a doctor.

Type 2: Any obvious active bleeding that has not yet met the following criteria for types 3-5, but meets at least one of the following conditions: (a) requires medical intervention; (b) requires hospitalization or increased level of treatment; (c) requires rapid evaluation.

Type 3

Type 3a: significant bleeding leading to a decrease in hemoglobin level of 3~5g/dl (30~50g/L); significant bleeding requiring blood transfusion.

Type 3b: significant bleeding leading to a decrease in hemoglobin level of ≥5g/dl (50g/L); cardiac tamponade; bleeding requiring surgical intervention or control (except for teeth, nose, skin, and hemorrhoids); intravenous vasoactive drugs are required.

Type 3c intracranial hemorrhage (excluding microbleeds, hemorrhagic transformation, including intraspinal hemorrhage); subtypes confirmed by autopsy, imaging examination, and lumbar puncture; intraocular hemorrhage that damages vision.

Type 4: intracranial hemorrhage within 48 hours of the perioperative period related to CABG-related bleeding; reoperation is required to control bleeding after sternotomy; more than 5U of whole blood or concentrated red blood cells need to be transfused within 48 hours; more than 2L of thoracic duct drainage within 24 hours.

Type 5: fatal bleeding

Type 5a: possible fatal bleeding; not confirmed by autopsy or imaging examination, but clinically suspected.

Type 5b: definite fatal bleeding; obvious or confirmed by autopsy and imaging examination50g /L).

2. The diagnostic criteria for clinical diseases during the patient's hospitalization are as follows:

1) Hypertension: Hypertension can be diagnosed if the patient has a previous diagnosis of hypertension or exhibits a systolic blood pressure of 140mmHg or higher and/or a diastolic blood pressure of 90mmHg or higher or is undergoing antihypertensive treatment.

2) Diabetes: Diabetes diagnosis can be established if the patient has a previous diagnosis of diabetes, is undergoing hypoglycemic treatment, or exhibits fasting blood glucose levels ≥ 123mg/dL and 2-hour blood glucose levels ≥ 200mg/dL as per an oral glucose tolerance test, or presents with typical symptoms accompanied by blood glucose levels ≥ 200mg/dL or has a glycated hemoglobin level＞6.5%.

3) Abnormal blood lipids: The diagnosis of hyperlipidemia can be made if the patient has a history of hyperlipidemia, is currently receiving lipid-lowering treatment, and meets the diagnostic criteria for hyperlipidemia at initial diagnosis (total cholesterol ≥ 220mg/dL, triglycerides ≥ 150mg/dL, low-density lipoprotein cholesterol ≥ 140mg/dL, high-density lipoprotein cholesterol ≤ 40mg/dL).

4) Chronic kidney disease (CKD): Chronic kidney disease is characterized by structural and functional impairment of the kidneys for a period exceeding 3 months, including pathological damage to normal and abnormal glomerular filtration rate, abnormal blood or urine composition, imaging abnormalities, or an unexplained decrease in glomerular filtration rate (＞60ml/min·1.73 m^2^).

5) Smoking: A positive smoking status is indicated if the patient has smoked in the past month, with a quantity exceeding 20.

6) Drinking: Alcohol consumption is considered positive if the patient has consumed alcohol in the past month, with a pure alcohol amount of 125 mg/d or approximately 150 g/d of Baijiu, or more than 4 bottles of beer per day.

7) Family history of coronary heart disease: A positive family history of coronary heart disease is established if a first-degree relative has been diagnosed with coronary heart disease before the age of 55 (male relative) or 65 (female relative).

3. Diagnostic criteria of OCT image:

Vulnerable plaque is unstable and prone to rupture, also known as "criminal plaque". Its diagnosis needs to be based on electrical changes, echocardiography results, and coronary angiographic results. Specifically, its main pathological characteristics are thin fibrous cap atherosclerotic plaque, large lipid rich necrotic core (LRNC), and macrophage infiltration. OCT defines a nonculprit plaque by the disappearance of the normal lumen’s three-layer structure and a stenotic area >30% greater than the diameter of the reference vessel (3). Lipid core length was defined as the length of a plaque with a lipid arc >90° measured on the longitudinal view. The lipid index was defined as the lipid core length multiplied by the mean lipid arc. The fibrous cap thickness of each lipid-rich plaque was measured three times on the thinnest part, and the mean value was taken. Calcified plaques are signal-poor regions with clear boundaries on OCT images. Fibrous plaques are homogeneous high-reflectivity and low-attenuation regions on OCT images. Lipid-rich plaques are signal-poor regions with diffuse boundaries, and the homogeneous signal-rich bands covering the surfaces of these signal-poor regions are called fibrous caps. The characteristics of macrophages are highly reflective, strongly attenuated point like or strip like structures, and often form radiating light shadows behind high signal point like areas. Generally speaking, macrophages typically aggregate at the boundaries of fiber caps and lipid pools. Lipid arcs were measured every 1 mm in the entire lesion, and the mean and maximum lipid arcs were recorded. Microcalcifications were signal-poor regions with clear boundaries, with an arc ≤90° within the cross-section and a length of ≤4 mm within the longitudinal section (4). PR was defined as discontinuity of the fibrous cap, with communication between vascular lumens (5).Thrombus was defined as a mass-like structure with a diameter ≥250 μm attached on the lumen surface or protruding into the lumen (6, 7).

All analysis and measurement methods for OCT images were consistent with previous diagnostic criteria (6,8).

4. The exclusion criteria

The exclusion criteria included a history of myocardial infarction before the ischemic stroke, coronary stenting or coronary artery bypass graft surgery, cardiogenic shock, severe renal and/or liver dysfunction, allergy to contrast agents, contraindications to antithrombotic therapy, left main vessel disease, chronic complete occlusion, severe tortuosity and calcification of blood vessels that would prevent the passage of an OCT guidewire, a high burden of thrombi. And poor OCT imaging quality unsuitable for analysis were also excluded.

5. Supplementary Tables

**Table S1** Comparison of risk factors for clinical data between non-stroke group and stroke group before PSM.

| Variables | Stroke group  (n = 193) | Nonstroke group  (n = 857) | *P*-value |
| --- | --- | --- | --- |
| Age, years | 65.0 (58.0, 72.0) | 60.0 (51.0, 67.0) | <0.001 |
| Male | 139 (72.0) | 618 (72.1) | 0.980 |
| Hypertension | 127 (65.8) | 377 (44.0) | <0.001 |
| Diabate Mellitus | 52 (26.9) | 176 (20.5) | 0.051 |
| Dyslipidemia | 51 (26.4) | 191 (22.3) | 0.218 |
| Previous CHD | 51 (26.4) | 265 (30.9) | 0.219 |
| Current smoker | 114（59.1） | 557（65.3） | 0.007 |
| Current drinker | 55（28.5） | 166（19.4） | <0.001 |
| BMI | 25.3 (22.6, 27.8) | 25.0 (22.8, 27.6) | 0.551 |
| Laboratory data |  |  |  |
| HbA1c, % | 6.4 ± 1.3 | 6.4 ± 2.0 | 0.968 |
| Hs-CRP, mg/L | 7.6 ± 5.2 | 6.3 ± 5.1 | <0.001 |
| TC, mg/dL | 166.5 ± 55.4 | 177.7 ± 45.4 | 0.003 |
| TG, mg/dL | 138.5 ± 86.2 | 151.1 ± 114.0 | 0.147 |
| HDL-C, mg/dL | 45.7 ± 14.7 | 44.5 ± 9.9 | 0.293 |
| LDL-C, mg/dL | 98.9 ± 39.8 | 108.8 ± 41.4 | 0.002 |
| EF, % | 61.0 (54.0, 62.0) | 61.0 (56.0, 62.0) | 0.447 |
| NT-pro BNP, pg/mL | 1331.5 (506.5, 3398.5) | 884.0 (307.0, 1909.0) | <0.001 |
| Previous medication use |  |  |  |
| Statins | 49 (25.4) | 108 (12.6) | <0.001 |
| ACEI | 26 (13.5) | 53 (6.2) | <0.001 |
| β-Blocker | 36 (18.7) | 65 (7.6) | <0.001 |
| CCB | 46 (23.8) | 124 (14.5) | 0.001 |

Note: Data are presented as median (interquartile range) or number (%)

ACEI — angiotensin-converting enzyme inhibitors; ApoA — Apolipoprotein A; ApoB — Apolipoprotein B; ARB — angiotensin receptor blocker; CAD — coronary artery disease; CCB — calcium channel blockers; CKD — Chronic kidney disease; Cr — creatinine; HbAlc — hemoglobinA1c; HDL-C — high-density lipoprotein cholesterol; hs-CRP — high-sensitive C-reactive protein; LDL-C — low-density lipoprotein cholesterol; TC — total cholesterol, TG — Triglyceride

**Table S2** Univariate analysis and multivariate logistic regression between stroke and clinical outcomes

| Variables | Univariate analysis | |  | Multivariate analysis | |
| --- | --- | --- | --- | --- | --- |
|  | *P* value | OR (95%CI) |  | *P* value | OR (95%CI) |
| Recurrent stroke | 0.026 | 4.28 (1.19, 15.42) |  | 0.016 | 4.87 (1.35, 17.61) |
| Cardiogenic death | 0.018 | 4.66 (1.31, 16.63) |  | 0.009 | 5.49 (1.52, 19.75) |
| Death for other causes | 0.332 | 3.08 (0.32, 29.91) |  | 0.211 | 4.81 (0.41, 56.37) |
| Recurrent MI | 0.642 | 1.53 (0.25, 9.28) |  | 0.692 | 1.50 (0.20, 11.20) |
| Readmission for angina or | 0.163 | 1.95 (0.76, 5.01) |  | 0.196 | 1.96 (0.71, 5.42) |
| heart failure |  |  |  |  |  |
| Revascularization | 0.239 | 2.08 (0.62, 7.03) |  | 0.483 | 1.67 (0.40, 6.97) |
| Bleeding | 0.982 | 1.02 (0.25, 4.13) |  | 0.538 | 0.60 (0.12, 3.08) |

MACCEs — Major adverse cardiac and cerebrovascular event; MI — Myocardial infarction

**References**

1. Gladstone DJ, Lindsay MP, Douketis J, Smith EE, Dowlatshahi D, Wein T, et al. Canadian Stroke Best Practice Recommendations: Secondary Prevention of Stroke Update 2020. *Can J Neurol Sci*. (2022) 49(3):315-337. doi: 10.1017/cjn.2022.15

2. Roy-O'Reilly M, McCullough LD. Age and Sex Are Critical Factors in Ischemic Stroke Pathology. *Endocrinology*. (2018) 159(8):3120-3131. doi: 10.1210/en.2018-00465

3. Kato K, Yonetsu T, Jia H, Abtahian F, Vergallo R, Hu S, et al. Nonculprit coronary plaque characteristics of chronic kidney disease. *Circ Cardiovasc Imaging*. (2013) 6(3):448-56. doi: 10.1161/CIRCIMAGING.112.000165

4. Mehran R, Rao SV, Bhatt DL, Gibson CM, Caixeta A, Eikelboom J, et al. Standardized Bleeding Definitions for Cardiovascular Clinical Trials: A Consensus Report From the Bleeding Academic Research Consortium. *Circulation*. (2011) 123(23):2736-2747. doi: 10.1161/CIR.0b013e31821b72c5

5. Xu G, Ma M, Liu X, Hankey GJ. Is there a stroke belt in China and why? *Stroke*. (2013) 44(7):1775-83. doi: 10.1161/STROKEAHA.113.001238

6. Tearney GJ, Regar E, Akasaka T, Adriaenssens T, Barlis P, Bezerra HG, et al. Consensus standards for acquisition, measurement, and reporting of intravascular optical coherence tomography studies: a report from the International Working Group for Intravascular Optical Coherence Tomography Standardization and Validation. *J Am Coll Cardiol*. (2012) 59(12):1058-72. doi: 10.1016/j.jacc.2011.09.079

7. Jang IK, Tearney GJ, MacNeill B, Takano M, Moselewski F, Iftima N, et al. In vivo characterization of coronary atherosclerotic plaque by use of optical coherence tomography. *Circulation*. (2005) 111(12):1551-5. doi: 10.1161/01.CIR.0000159354.43778.69

8. Virmani R, Burke AP, Farb A, Kolodgie FD. Pathology of the vulnerable plaque. *J Am Coll Cardiol*. (2006) 47(8 Suppl):C13-8. doi: 10.1016/j.jacc.2005.10.065
